# A Case of Grade 4 Hyperglycemia Induced by Capivasertib and Successfully Managed with Early Insulin Intervention

**DOI:** 10.70352/scrj.cr.25-0514

**Published:** 2025-12-11

**Authors:** Yuki Nomoto, Miki Mukai, Yuka Eguchi, Heiji Yoshinaka, Yoshiaki Shinden, Akihiro Nakajo

**Affiliations:** 1 Department of Breast Surgery, Kagoshima City Hospital, Kagoshima, Kagoshima, Japan; 2 Department of Breast and Thyroid Surgery, Kagoshima University Graduate School of Medical and Dental Sciences, Kagoshima, Kagoshima, Japan; 3 Department of Diabetes, Metabolism and Endocrinology, Kagoshima City Hospital, Kagoshima, Kagoshima, Japan

**Keywords:** capivasertib, hyperglycemia, insulin resistance, breast cancer

## Abstract

**INTRODUCTION:**

Capivasertib, a protein kinase B (AKT) inhibitor, has demonstrated robust efficacy against hormone receptor–positive, human epidermal growth factor receptor type 2–negative advanced breast cancer, particularly in patients with AKT pathway mutations. However, serious metabolic complications such as hyperglycemia and diabetic ketoacidosis (DKA) have been reported. This case report describes capivasertib-induced hyperglycemia to support the development of safe treatment and monitoring strategies. The case is notable because early detection and timely intervention following the rapid onset of grade 4 hyperglycemia resulted in a favorable clinical course without long-term glycemic complications.

**CASE PRESENTATION:**

A 69-year-old woman without diabetes developed grade 4 hyperglycemia (522 mg/dL) on day 2 after receiving capivasertib in combination with fulvestrant therapy. Pretreatment glucose tolerance test results were normal. Continuous insulin infusion was promptly initiated in response to the rapid increase in blood glucose levels to prevent ketosis. Her blood glucose levels improved within half a day, and she was discharged on day 6. She did not require further insulin or hypoglycemic agents thereafter.

**CONCLUSIONS:**

Inpatient glucose monitoring enabled prompt detection of rapid glucose elevation and early intervention before the onset of DKA, resulting in a favorable outcome. Further clinical evidence is required to define optimal intervention strategies for grade 4 hyperglycemia associated with capivasertib therapy.

## Abbreviations

AKT
: protein kinase B
DKA
: diabetic ketoacidosis
GLUT-4
: glucose transporter type 4
GSK3
: glycogen synthase kinase-3
HER2
: human epidermal growth factor receptor type 2
mTOR
: mammalian target of rapamycin
PI3K
: phosphoinositide 3-kinase

## INTRODUCTION

Capivasertib is a selective AKT inhibitor that targets the PI3K/AKT/mTOR signaling pathway, which plays a key role in tumor cell growth and survival. The CAPItello-291 trial demonstrated that capivasertib combined with fulvestrant significantly prolonged progression-free survival in patients with hormone receptor–positive, HER2–negative advanced breast cancer, particularly in those with PIK3CA, AKT1, or PTEN mutations.^1)^ However, frequent adverse events such as diarrhea, rash, and hyperglycemia have been reported, with hyperglycemia occasionally progressing to DKA. In Japan, postmarketing surveillance identified DKA–related deaths, prompting the Japan Diabetes Society to issue a safety alert in April 2025 regarding the risk of hyperglycemia and DKA associated with capivasertib.^2)^ We present a case of grade 4 hyperglycemia in a patient without diabetes who was receiving capivasertib, successfully managed with early insulin intervention before the onset of DKA.

## CASE PRESENTATION

A 69-year-old woman (height: 141.9 cm; weight: 46.6 kg) with advanced breast cancer complicated by multiple bone and lung metastases initiated first-line therapy with fulvestrant plus abemaciclib in October 2021. Owing to disease progression in January 2025, she was treated with epirubicin and cyclophosphamide as second-line therapy while awaiting the results of FoundationOne CDx (Foundation Medicine, Inc., Cambridge, MA, USA) testing, which revealed a PTEN mutation. Consequently, third-line therapy with capivasertib and fulvestrant was initiated.

We consulted a diabetologist prior to initiating therapy, who recommended a comprehensive pretreatment evaluation that revealed no evidence of glucose intolerance (**Tables 1** and **2**). Capivasertib therapy was initiated during hospitalization, with 6-point daily glucose monitoring performed. On day 2, at approximately 7:00 AM, the patient reported urinary frequency and dysuria. The patient’s fasting blood glucose level was 92 mg/dL. Urinalysis revealed pyuria, hematuria, and proteinuria, but no glucosuria or ketonuria. Based on these findings, hyperglycemia was ruled out, and a diagnosis of cystitis was made. Capivasertib (200 mg; two tablets) was administered at 8:30 AM. Two hours later, the patient’s postprandial glucose level was 193 mg/dL. The glucose level increased to 219 mg/dL before lunch and peaked at 443 mg/dL after lunch. As this value met the criteria for grade 3 hyperglycemia, a diabetologist was consulted. Glucose levels continued to rise, reaching 471 mg/dL at 3:30 PM and 522 mg/dL at 4:30 PM. Following consultation, she was admitted to the ICU at 5:00 PM, where a continuous intravenous insulin infusion was initiated at a rate of 10 units/h. Her blood glucose level gradually decreased, and the infusion was discontinued at 11:00 PM on the same day. The patient was transferred from the ICU to the general ward at 10:30 AM on day 3 (**Table 3**). On days 4 and 5, 6-point daily glucose monitoring was continued, and stable blood glucose levels were observed. The patient was discharged on day 6 without the need for insulin or other hypoglycemic agents. This episode was classified as grade 4 hyperglycemia, and capivasertib was subsequently discontinued.

**Table 1:** Pre-treatment glucose evaluation

Test item	Value
HbA1c	5.20%
Glycated albumin	13.60%
Anti-GAD antibody	<0.5 U/mL

Pre-treatment glucose evaluation included HbA1c, glycated albumin, and anti-GAD antibody levels. All values were within the normal range, indicating no evidence of pre-existing glucose intolerance prior to initiating capivasertib.

Anti-GAD, anti-glutamic acid decarboxylase; HbA1c, hemoglobin A1c

**Table 2:** Six-point daily glucose profile (prior to treatment)

Time	Blood glucose level (mg/dL)
Before breakfast	92
After breakfast	134
Before lunch	108
After lunch	132
Before dinner	105
After dinner	128

Six-point daily glucose monitoring was performed prior to initiating capivasertib. Blood glucose levels were measured before and 2 h after each main meal (breakfast, lunch, and dinner) to confirm normal glucose tolerance before therapy initiation

**Table 3:** Blood glucose levels, urinary findings, and laboratory data during the first 3 days of capivasertib treatment

	Day 1						Day 2					
	7:00	10:00	11:00	14:00	17:00	20:00	7:00	10:00	11:00	14:00	15:30	16:30
Blood glucose level (mg/dL)	90	119	161	184	70	156	92	193	219	443	507	522
Immunoreactive insulin (μU/mL)	7.1										4760	
Continuous intravenous insulin infusion (U/h)												
Ketonuria								(−)				
Glucosuria								(−)				
pH											7.385	7.39
pCO_2_ (mmHg)											35.7	33.2
pO_2_ (mmHg)											66.6	86.4
cHCO_3_^−^ (mmol/L)											20.9	19.7
cBase (mmol/L)											−3.2	−4.2
cNa^+^ (mmol/L)											133	134
cK^+^ (mmol/L)											4.2	4.2
Lac (mmol/L)											1.1	1.7

	Day 2						Day 3						
	17:00	18:30	18:00	19:00	20:30	23:00	2:00	5:00	7:00	9:00	12:00	17:00	20:00
Blood glucose level (mg/dL)	475		423	372	294	123	84	107	160	81	113	98	120
Immunoreactive insulin (μU/mL)											19.5		
Continuous intravenous insulin infusion (U/h)	10		5	2	1	OFF							
Ketonuria	(−)		(−)								(−)		
Glucosuria	(4+)		(4+)								(−)		
pH	7.393	7.387	7.404	7.392	7.381	7.415	7.414	7.446	7.452	7.466			
pCO_2_ (mmHg)	32.8	34	34.5	36.1	37.1	35.7	36.3	33.3	32.8	32.5			
pO_2_ (mmHg)	101	91	93.7	93.3	97.8	82.7	91.9	104	101	101			
cHCO_3_^−^ (mmol/L)	19.5	20	21.2	21.5	21.5	22.4	22.8	22.5	22.5	23.2			
cBase (mmol/L)	−4.3	−4	−2.7	−2.6	−2.7	−1.4	−1.1	−0.8	−0.7	0.1			
cNa^+^ (mmol/L)	134	133	133	135	137	137	138	139	138	137			
cK^+^ (mmol/L)	4	4	3.8	3.7	3.6	3.1	3.2	3.5	3.4	3.5			
Lac (mmol/L)	1.3	1	1	1.1	1.6	1.1	0.6	0.7	1.3	0.6			

Blood glucose levels, urinary ketone bodies (ketonuria), and urinary glucose (glucosuria) were monitored 6 times daily from days 1 to 3 after starting capivasertib. Immunoreactive insulin levels were measured at baseline, at the peak of hyperglycemia (day 2), and after glucose normalization (day 3). Continuous intravenous insulin infusion rates (U/h) are provided for each time point in the ICU. Arterial blood gas analysis included pH, pCO_2_, pO_2_, cHCO_3_^+^, cBase, cNa^+^, cK^+^, and Lac levels. The peak blood glucose level of 522 mg/dL occurred on day 2, followed by rapid improvement after insulin initiation.

cBase, base excess; cHCO_3_^−^, bicarbonate; cK^+^, potassium; cNa^+^, sodium; Lac, lactate; pCO_2_, partial pressure of carbon dioxide, pO_2_, partial pressure of oxygen

## DISCUSSION

Capivasertib, a selective oral AKT inhibitor, has demonstrated substantial efficacy in hormone receptor–positive, HER2–negative advanced breast cancer, particularly among patients with AKT pathway mutations. In the CAPItello-291 trial, it significantly improved progression-free survival.^1)^

The PI3K/AKT/mTOR pathway plays a central role in cellular metabolism, growth, survival, and proliferation by transmitting extracellular signals through a kinase cascade. Alterations in this pathway are found in up to 70% of breast cancers, leading to hyperactivation that promotes tumor initiation, proliferation, and resistance to apoptosis.^3)^ In skeletal muscle, insulin binding to its receptor activates PI3K–AKT, promoting GLUT-4 translocation and glucose uptake. In the liver, this pathway inhibits GSK3, activates glycogen synthase, and promotes glycogen storage. Capivasertib blocks AKT activity in these tissues, impairing glucose uptake and glycogen synthesis and ultimately leading to hyperglycemia.^4,5)^ Type 1 diabetes is caused by autoimmune β-cell destruction,^6)^ whereas type 2 diabetes is characterized by insulin resistance.^7)^ Thus, capivasertib-induced hyperglycemia more closely resembles type 2 diabetes.

In the CAPItello-291 trial, hyperglycemia was reported in 16.9% of patients (grade ≥3 in 2.3%), typically within the first two weeks (median onset, 15 days).^1)^ Most cases were transient and manageable with short-term insulin or metformin use in affected patients.^8)^ Similarly, in Japanese postmarketing surveillance, which included approximately 350 patients, 31 cases of hyperglycemia and six acute hyperglycemic complications (including DKA and hyperosmolar hyperglycemic syndrome) were reported. One patient died of DKA. Most cases of hyperglycemia developed within the first two weeks of treatment; of these, 17 patients (46%) recovered and 5 (14%) improved.^9)^

Consistent with this mechanism, our patient’s immunoreactive insulin level at the time of peak hyperglycemia was 4760 μU/mL, markedly higher than the baseline value of 7.1 μU/mL. It decreased to 19.5 μU/mL by day 3, suggesting severe but reversible insulin resistance rather than insulin deficiency. This finding further supports that capivasertib-induced hyperglycemia represents a reversible form of insulin resistance.

Other inhibitors targeting the PI3K/AKT/mTOR pathway have also been associated with hyperglycemia as an on-target class effect owing to interference with insulin signaling.^3)^ Hyperglycemia has also been reported with the AKT inhibitor ipatasertib in the phase III IPATunity130 trials for triple-negative and hormone receptor–positive/HER2–negative breast cancer (any grade, 14%–19%; grade ≥3, ≤2%), with similar rates in the placebo arms.^10,11)^ In contrast, the PI3K inhibitor alpelisib caused grade ≥3 hyperglycemia in 36% of patients in the SOLAR-1 trial, which was usually managed with metformin or insulin and resolved within a median of six days.^12)^ The mTOR inhibitor everolimus also induced hyperglycemia in 13% of patients (grade ≥3 in ~5%).^13)^

Two previous case reports have documented severe DKA associated with capivasertib in patients without pre-existing diabetes. Rodriguez et al. described a 77-year-old woman with metastatic breast cancer who developed profound hyperglycemia (718 mg/dL) and DKA 10 days after treatment initiation, despite a normal baseline HbA1c. Her condition was refractory to standard-dose insulin infusion and deteriorated owing to renal failure.^14)^ Habeb et al. reported a 71-year-old man with metastatic prostate cancer who developed DKA six months after starting capivasertib. He presented with polyuria, polydipsia, and confusion, required prolonged insulin infusion for glucose stabilization, and was discharged on basal-bolus insulin therapy after drug discontinuation.^15)^ These reports indicate that, although rare, AKT-inhibitor–induced hyperglycemia can occasionally present as life-threatening DKA, underscoring the need for early detection and prompt management.

In our case, the initial insulin infusion rate of 10 units/h exceeded the standard DKA protocol (0.1 U/kg/h),^16)^ reflecting a proactive response based on previous Japanese cases of capivasertib-related DKA. This approach, guided by early specialist input, likely facilitated the prompt resolution of hyperglycemia.

At our institution, capivasertib had previously been initiated in the outpatient setting. However, this case occurred immediately after the nationwide safety alert issued by the Japan Diabetes Society regarding the risk of hyperglycemia and DKA. Because the patient lived alone and had limited access to immediate medical care in the event of hyperglycemic symptoms, inpatient initiation was chosen to ensure safety.

Based on this experience, our institution has adopted a risk-adapted approach. During pre-treatment counseling, patients are thoroughly informed about the potential adverse effects of capivasertib, including hyperglycemia and DKA, so that they can understand the risks and participate in treatment decisions. Those who wish may begin treatment with short-term hospitalization (approximately one week) for safety monitoring. For patients who prefer outpatient initiation, we instruct them to pay close attention to early symptoms of hyperglycemia—such as polyuria, dysuria, polydipsia, or dry mouth—and to promptly visit the hospital for intensive glucose monitoring if such symptoms occur. In our case, an early clinical clue was noted: on the morning of day 2, the patient reported urinary frequency and dysuria before any clear biochemical evidence of hyperglycemia was present. Neither blood glucose levels nor urinalysis findings at that time predicted the subsequent development of severe hyperglycemia.

With further case accumulation, we aim to clarify the optimal timing for diabetology consultation and intervention, and to determine whether hyperglycemia develops with symptoms or silently. Ultimately, we seek to establish a safe and practical outpatient initiation protocol for capivasertib through risk-stratified monitoring and patient education.

## CONCLUSIONS

This case demonstrates that, even in non-diabetic patients, capivasertib can lead to severe hyperglycemia, and early detection combined with prompt initiation of continuous insulin infusion is highly effective. Multidisciplinary collaboration, including consultation with a diabetologist, is essential to ensure the safe administration of capivasertib.

## DECLARATIONS

### Availability of data and materials

The datasets supporting the conclusions of this article are included within the article.
